# Supplementary material for: From dyadic coping to emotional sharing and multimodal interpersonal synchrony: Protocol for a laboratory experiment
Source: PLoS One. 2025 May 20;20(5):e0323526. doi: 10.1371/journal.pone.0323526 (PMC12091729; doi:10.1371/journal.pone.0323526)
Supplement: S1 File — (DOCX) [file pone.0323526.s001.docx]

**Supplementary 1: Proposed Experiment and Hypotheses**

**Main Hypotheses**

At the *phasic timescale*, we will examine the emergence of spontaneous synchronization in relationship partners’ a) movements (Koole & Tschacher, 2016) and a) cardiovascular responding (Palumbo et al., 2017). Movement synchrony indexes fluency of interaction and bonding (Scheidt et al., 2021). Accordingly, we predict that relationship partners will display statistically significant levels of movement synchrony during the experiment, relative to a relevant baseline of randomized pseudo-interactions (Refs to Ramseer & Tschacher). Cardiovascular synchrony indexes mutual regulation of emotional arousal (Palumbo et al., 2017). More specifically, our cardiovascular assessment allows us to assess Pre-Ejection Period (PEP) as a marker of sympathetic nervous activity and Respiratory Sinus Arrhythmia (RSA) as a marker of parasympathetic nervous activity. Across both sympathetic and parasympathetic markers, we predict that relationship partners will display statistically significant levels of cardiovascular synchrony during the experiment, relative to a relevant baseline of randomized pseudo-interactions (Ramseyer & Tschacher, 2010).

At the *tonic timescale*, we predict that emotional sharing will generally lead sharers to feel somewhat better, leading to positive mood changes, and to a somewhat better understanding of their negative experiences leading to sharing-induced changes in emotional appraisals. We further predict that the effects of emotional sharing will be moderated by instructed co-rumination (versus natural sharing. More specifically, we predict that instructed co-rumination will make the sharers feel better, leading them to report positive mood changes. At the same time, we predict that co-rumination will interfere with considering new perspectives on the emotion-eliciting situation. Thus, we predict that instructed co-rumination will lead to less sharing-induced changes in emotional appraisals. We further predict that instructed co-rumination will have systematic effects on interpersonal synchronization processes. First, we predict that engaging in emotional sharing will lead to increases in movement synchrony and cardiovascular synchrony (across both sympathetic and parasympathetic markers). Second, we predict that these increases will be bolstered by co-rumination. Thus, sharing-induced increases in movement synchrony and cardiovascular synchrony are expected to be greater when participants are instructed to engage in co-rumination versus natural sharing.

At the *chronic timescale*, our general expectation is that chronic individual dispositions and relationship patterns will display meaningful associations with phasic and tonic processes during the emotional sharing task. We predict that superior and positive dyadic coping strategies, coupled with higher relationship quality between partners, will give rise to increased levels of movement and cardiovascular synchrony (across both sympathetic and parasympathetic markers).

**Exploratory Analyses**

Because of the innovative nature of the planned study, there are many new things that we hope to learn from it in a more exploratory manner, without have a strong rationale from the existing literature in interpersonal emotion regulation. In what follows, we outline our main plans for these exploratory analyses. It's expected that these plans may undergo revisions as we gain further insights into the project.

**1)** **Linguistic Synchrony:** We intend to conduct a detailed analysis of the content of participants' conversations. Coding will include an examination of the frequency and nature of specific words used during emotional sharing, with a focus on identifying patterns associated with co-rumination and chronic variables. Of particular interest are the use of insight words and linguistic synchrony.

Prior work on written emotional disclosure has found that changes in words suggestive of causal and insightful thinking were linked to positive health change (Pennebaker & Francis, 1996). Causal thinking involves considering the cause-and-effect relationships between events or emotions and insightful thinking also involves gaining a deeper, more perceptive understanding of one's emotions or experiences. Therefore, individuals who demonstrated shifts in their language towards more causal and insightful thinking during the process of emotional disclosure experienced positive changes in their health. we predict that a greater use of vocabulary implying causal relationships or insight during the emotional sharing process will be associated with more significant changes in emotion and appraisal, with a stronger correlation in co-rumination situations.

Prior research has found evidence for spontaneous matching of words in face-to-face interaction, a phenomenon known as linguistic synchrony (Doré & Morris, 2018; Ireland & Pennebaker, 2010; Ireland et al., 2011; Tay & Qiu, 2022). linguistic synchrony refers to the coordination and alignment of language, particularly verbal communication, between individuals engaged in an emotional sharing task within close relationships. As evidenced in the field of psychotherapy, we predict that higher levels of linguistic coordination and alignment are associated with greater emotional changes, potentially influencing chronic processes (personal dispositions and relationship patterns).

**2) Vocal synchrony:** Vocal expressions can convey emotional arousal and intensity during interpersonal interactions (Reich et al., 2014; Schoenherr et al., 2021). We will code vocalizations for signs of arousal, pitch variations, and other acoustic features that may be indicative of emotional states. Exploring the relationship between vocal arousal and other measures (e.g., movement synchrony, cardiovascular synchrony) will contribute to a more comprehensive understanding of emotional expression and regulation within close relationships.

**3)** **Facial Synchrony:** Research indicates that the social context of sharing emotions between individuals exhibits features of neural, autonomic, and facial synchrony (Altmann et al., 2021; Butler, 2015; Golland et al., 2015). It is well-established that people mimic the facial expressions of others, a behavior that can facilitate the understanding of others' feelings and emotional contagion (Hess & Fischer, 2014; Prochazkova & Kret, 2017). A recent study showed that co-present individuals often become synchronized in facial dynamics, and this facial synchrony is associated with emotional similarity (Golland et al., 2019).

We will employ facial expression coding to analyze emotional expressions displayed by participants during the emotional sharing task. This analysis will help identify the emotional dynamics at play during the task, and we will explore whether facial expressions align with other measures of emotional sharing, such as movement synchrony and cardiovascular synchrony.

**Supplementary 2: Measurement**

**Questionnaires Measures**

**Tonic Timescale**

The questionnaires for the tonic timescale will assess several aspects of participants’ experience before, during, and after the emotional sharing task.

*The Mood Adjective Checklist (BEF)*

The BEF is an extended version of the Positive and Negative Affect Schedule (PANAS; see Watson et al., 1988). The items are assessed using 4-point Likert scales, ranging from 0 (not at all) to 3 (completely). The inventory consists of seven subscales: 1) Pleasantness, 2) Activation, 3) Relaxation, 4) Helplessness, 5) Distress, 6) Listlessness, and 7) Anger. We will combine the pleasantness, activation, and relaxation scales into a measure of positive affect, and we will combine the helplessness, distress, listlessness, and anger scales into a measure of negative affect.

*Experience Questionnaire (EQ)*

The ES assesses participants’ emotional appraisal of the negative experiences that constitute the focal topics of the emotional sharing task. The EQ has two parts: the first part inquiries about the emotions evoked by the experiences, such as sadness, shame, guilt etc. The second part involves a modified version of the general appraisal questionnaire (GAQ, Scherer, 2001), primarily assessing three key domains: 1) Intensity and duration of negative emotions (e.g., How intense were the negative emotions that you had during this experience? Four items); 2) To what extent do you think that one or more of the following factors led you to have the experience? (e.g., The behavior of one or more other person, to what extent did they cause it intentionally? Three items); 3) With regard to the actual or potential consequences of the event, (To what extent have these already been felt by you? Nine items).

*Inclusion of Other in the Self Scale (IOSS)*

The inclusion of other in the self scale (Aron et al., 1992) will be used to assess how close the respondent feels with another person. The individual items of the IOS scale consist of seven images, each depicting two overlapping circles. These images, progressing from the first to the seventh, represent a gradual increase in the degree of overlap between the two circles. Respondents intuitively perceive the degree of overlap as an indicator of the closeness of the relationship between the themes depicted within the circles, such as the relationship between the respondent and the "other" identified within the circles; a higher degree of overlap signifies a closer relationship. Participants are instructed to identify which of the seven images best represented their relationship with the "other".

*Sharer/Listeners’ Own Experience (SOE)*

After each sharing episode, the experiences of both the sharer and the listener in emotion-sharing conversation will be individually assessed. The wording of the six items, ranging from 1 (total disagreement) to 7 (total agreement), is such that they form parallel questions for the sharer and the listener. For instance, the sharer will be asked to rate the item, "I felt completely like myself during the conversation", whereas the listener will be asked to rate the item, "My partner felt completely like himself during the conversation."

*Perceived Partner Responsiveness (PPR)*

Each participant's perception of their partner's responsiveness will be assessed using three items adapted from Reis (2012), rated on a scale from 1 (total disagreement) to 7 (total agreement). These items include statements like 'My partner understood me,' 'My partner cared for me,' and 'My partner appreciated who I really am.' Similarly, the listener's perception of their partner's responsiveness will be measured using three items, also rated on a scale from 1 to 7, including statements such as 'I understood my partner,' 'I cared for my partner,' and 'I appreciated who my partner really is.' Total scores are computed by summing the ratings for each item, with higher scores indicating a stronger perception of partner responsiveness.

*Interpersonal emotion regulation with* *different strategies from partner (IER-DSFP)*

Following each sharing episode, both the sharer and the listener will evaluate various interpersonal emotion regulation strategies (such as co-rumination, distraction, acceptance, ignoring) using a set of seven parallel items. Each item will be rated on a scale from 1 (strongly disagree) to 7 (strongly agree), allowing for comparative assessments between the sharer and the listener. For example, the sharer will rate the item, "My partner tried to get me to talk over and over about what is bothering me," while the listener will rate the item, "I tried to get my partner to talk over and over about what is bothering them."

*Sharing Quality (RQ)*

Four items will be used to measure different aspects of the perceived quality of emotional sharing. Specifically, the items will assess: 1) Agreement**:** ‘My partner and I agreed on how I could best approach the problem.’, from 1 (total disagreement) to 7 (total agreement); 2) Support: ‘How much did you feel supported by your partner?’, from 1 (Not at all) to 7 (Very Much); 3) Self-disclosure**:** ‘I could openly share and disclose all my thoughts and feelings with my partner’, from 1 (total disagreement) to 7 (total agreement); 4) Closeness**:** ‘How close you felt to your partner at this moment?’, from 1 (not close at all) to 7 (very close).

**Chronic Timescale**

The questionnaires for the chronic timescale will assess participants’ personality dispositions and various psychological dimensions of their close relationship.

*Toronto Alexithymia-20 Scale (TAS-20)*

The TAS-20 is the most widely used self-report measurement of alexithymia. It generally has good psychometric properties and validity (Bagby et al., 2020). The TAS-20 consists of 20 items rated on a 5-point scale from strongly disagree to strongly agree. It has three subscales: Difficulties in identifying feelings (DIF), e.g., “I am often confused about what emotion I am feeling”; Difficulties in describing feelings (DDF), e.g., “It is difficult for me to find the right words for my feelings”; and Externally oriented thinking (EOT), e.g., “I prefer to just let things happen rather than to understand why they turned out that way”. Both the complete scale and the subscales will be used in the analyses. We will use the Dutch version of the questionnaire (Kooiman et al., 2002).

*Partnership Questionnaire—Short Form (PQ-SF)*

The nine-item partnership questionnaire—short form (Kliem et al., 2015) is a reliable tool used to measure the quality of romantic relationships through three subscales: quarreling, tenderness, and togetherness/communication. Each of the three items in each subscale is rated on a four-point Likert scale (0 = never/very seldom to 3 = very often). The scores from each subscale can be combined to generate a total score ranging from 0 to 27, where higher scores indicate higher satisfaction.

*Partner-Specific Attachment Security Short Form (PSAS-SF)*

The partner-specific attachment security short form consists of 12 items (e.g., "It helps to turn to my partner in times of need") and primarily focused on adult attachment styles in the context of intimate relationships. All items are rated on a 7-point scale, ranging from 1 (strongly disagree) to 7 (strongly agree). The ECR-S contains two subscales assessing avoidant attachment and attachment anxiety (Wei et al., 2005).

*Dyadic Coping Inventory (DCI)*

The 41-item dyadic coping inventory measures the behaviors of one or both partners when they experience stress. The DCI has four subscales: 1) self-coping behavior (16 items, e.g., "I tell my partner that it is not that bad and help him/her to see the situation in a different light."), 2) partner’s coping behavior (16 items, e.g., "My partner takes on things that I normally do in order to help me out."), 3) common dyadic coping behavior (7 items, e.g., " We try to cope with the problem together and search for practical solutions"), and 4) overall satisfaction with dyadic coping (2 items, e.g., " I am satisfied with the support I receive from my partner and the way we deal with stress together."). All items use 4-point Likert scale (0 = never; 4 = very often; Bodenmann, 2008; Austin & Falconier, 2013).

*The Others and Self-Emotion Regulation Scale (OSRS)*

The 20-item others and self-emotion regulation scale will be utilized to assess participants' perception of emotion regulation (Niven et al., 2011). Participants are asked to assess how they handle the emotions of others (10 items, e.g., "I gave someone helpful advice to try to improve how my felt") and their own emotions (10 items, e.g., "I looked for problems in my current situation to make myself feel worse"). All items are rated on a 5-point scale, ranging from 0 (not at all) to 5 (great deal).

*Support Support List (SSL)*

The 12-item Social Support List (SSL-12) will be used to assess the level of perceived social support received, with response options ranging from 1 (seldom or never) to 4 (very often) The SSL-12 consists of three subscales: daily support, esteem support and support in problem situations. Higher scores indicate a higher perceived level of support (Kempen & Van Eijk 1995).

*Loneliness Scale (UCLA-LS)*

The 20-items University of California, Los Angeles (UCLA) loneliness scale (Russell, 1996) will be used to measure participants' subjective loneliness. The scale comprises 20 items, with 10 items worded positively and 10 items worded negatively, for instance, 'I lack companionship' or 'There are people I can talk to'. These items reflect satisfaction and dissatisfaction with social relationships. Participants are required to rate the frequency of several experiences on a 5-point scale, ranging from 1 (total disagreement) to 5 (total agreement).

*Interpersonal Reactivity Index (IRI)*

The 28-item interpersonal reactivity index (Davis et al., 1983) will be employed to measure various dimensions of empathy. The IRI ranges from 1 (does not describe me well) to 5 (describes me very well), designed to assess cognitive and emotional dimensions of empathy. This scale comprises four subscales, each containing 7 items, with each subscale pertaining to a specific aspect of the overall concept of empathy. The cognitive dimensions are assessed using the perspective taking (PT, e.g., "I sometimes find it difficult to see things from the other person's perspective.") and fantasy subscales (FS, e.g., "I regularly daydream and fantasize about things that could happen to me."), while the emotional dimensions are measured through the empathic concern (EC, e.g., "I am often very moved by things I see happen.") and personal distress (PD, e.g., "I tend to lose control in emergency situations.") subscales. Total scores for each subscale can range from 0 to 28.

*Distress Disclosure Index (DDI)*

The study will utilize the 12-item distress disclosure index (Kahn & Hessling, 2001) to assess participants' propensity to share personal distressing information. The DDI comprises items evaluating disclosure behaviors (e.g., " When I feel upset, I usually confide in my friends") and concealment behaviors (e.g., "I prefer not to talk about my problems"). Participants will rate their agreement level for each item on a scale ranging from 1 (strongly disagree) to 5 (strongly agree). Given that disclosure and concealment may occur simultaneously, reverse scoring will be applied to six concealment items, and scores will be aggregated, with higher scores indicating a greater inclination toward disclosure (Kahn & Hessling, 2001).

*Bem Sex Role Inventory (BSRI)*

The 20-item Bem sex role inventory (BSRI) is employed to assess individuals' identification with stereotypical masculine traits (BSRI-m, such as “assertive,” “forceful,” and “aggressive”) and stereotypical feminine traits (BSRI-f, such as “gentle,” “sympathetic,” and “loves children”). Participants are required to provide answers on a 7-point scale, ranging from "almost never or never true" to "almost always true."

*Sociodemographic Variables*

The following sociodemographic variables will be collected: age, sex assigned at birth (female/male), gender identity (female/male), residential environment (very urban, moderately urban, and little to non-urban) and educational level (primary education, lower /preparatory vocational education, general secondary education, secondary vocational education, higher general secondary education, preparatory scientific education, higher professional education, academic education, and other).

**References**

Aron, A., Aron, E. N., & Smollan, D. (1992). Inclusion of other in the self scale and the structure of interpersonal closeness. *Journal of personality and social psychology*, *63*(4), 596-612.

Austin, J. L., & Falconier, M. K. (2013). Spirituality and common dyadic coping: Protective factors from psychological aggression in Latino immigrant couples. *Journal of Family Issues*, *34*(3), 323-346.

Bagby, R. M., Parker, J. D., & Taylor, G. J. (2020). Twenty-five years with the 20-item Toronto Alexithymia Scale. *Journal of psychosomatic research*, *131*, 109940.

Bodenmann, G. (2008). Dyadic coping and the significance of this concept for prevention and therapy. *Zeitschrift für Gesundheitspsychologie*, *16*(3), 108-111.

Davis, D., Suppe, J., & Dahlen, F. A. (1983). Mechanics of fold‐and‐thrust belts and accretionary wedges. *Journal of Geophysical Research: Solid Earth*, *88*(B2), 1153-1172.

Kahn, J. H., & Hessling, R. M. (2001). Measuring the tendency to conceal versus disclose psychological distress. *Journal of Social and Clinical Psychology*, *20*(1), 41-65.

Kempen, G. I. J. M., & Van Eijk, L. M. (1995). The psychometric properties of the SSL12-I, a short scale for measuring social support in the elderly. *Social Indicators Research*, *35*, 303-312.

Kliem, S., Beller, J., Kröger, C., Stöbel-Richter, Y., Hahlweg, K., & Brähler, E. (2015). A Rasch re-analysis of the Partnership Questionnaire. *Sage Open*, *5*(2), 2158244015588958.

Kooiman, C. G., Spinhoven, P., & Trijsburg, R. W. (2002). The assessment of alexithymia: a critical review of the literature and a psychometric study of the Toronto Alexithymia Scale-20. *Journal of psychosomatic research*, *53*(6), 1083-1090.

Niven, K., Totterdell, P., Stride, C. B., & Holman, D. (2011). Emotion Regulation of Others and Self (EROS): The development and validation of a new individual difference measure. *Current Psychology*, *30*, 53-73.

Reis, H. T. (2012). *Perceived partner responsiveness as an organizing theme for the study of relationships and well-being*.

Russell, D. W. (1996). UCLA Loneliness Scale (Version 3): Reliability, validity, and factor structure. *Journal of personality assessment*, *66*(1), 20-40.

Scherer, K. R. (2001). Appraisal considered as a process of multilevel sequential checking.

Watson, D., Clark, L. A., & Carey, G. (1988). Positive and negative affectivity and their relation to anxiety and depressive disorders. *Journal of abnormal psychology*, *97*(3), 346-353.

Wei, M., Mallinckrodt, B., Larson, L. M., & Zakalik, R. A. (2005). Adult attachment, depressive symptoms, and validation from self versus others. *Journal of Counseling Psychology*, *52*(3), 368-377.
